# Presetting of the Corticospinal Excitability in the Tibialis Anterior Muscle in Relation to Prediction of the Magnitude and Direction of Postural Perturbations

**DOI:** 10.3389/fnhum.2019.00004

**Published:** 2019-01-17

**Authors:** Kimiya Fujio, Hiroki Obata, Noritaka Kawashima, Kimitaka Nakazawa

**Affiliations:** ^1^Department of Rehabilitation Science, Faculty of Health Care Science, Chiba Prefectural University of Health Sciences, Chiba, Japan; ^2^Department of Rehabilitation for the Movement Functions, Research Institute of the National Rehabilitation Center for Persons with Disabilities, Saitama, Japan; ^3^Department of Humanities and Social Sciences, Institute of Liberal Arts, Kyushu Institute of Technology, Fukuoka, Japan; ^4^Sports Science Laboratory, Department of Life Sciences, Graduate School of Arts and Sciences, University of Tokyo, Tokyo, Japan

**Keywords:** postural response, corticospinal pathway, prediction, transcranial magnetic stimulation, tibialis anterior muscle, motor preparation

## Abstract

The prediction of upcoming perturbation modulates postural responses in the ankle muscles. The effects of this prediction on postural responses vary according to predictable factors. When the amplitude of perturbation can be predicted, the long-latency response is set at an appropriate size for the required response, whereas when the direction of perturbation can be predicted, there is no effect. The neural mechanisms underlying these phenomena are poorly understood. Here, we examined how the corticospinal excitability of the ankle muscles [i.e., the tibialis anterior (TA), the soleus (SOL), and the medial gastrocnemius (MG), with a focus on the TA], would be modulated in five experimental conditions: (1) No-perturbation; (2) Low (anterior translation with small amplitude); (3) High (anterior translation with large amplitude); (4) Posterior (posterior translation with large amplitude); and (5) Random (Low, High, and Posterior in randomized order). We measured the motor-evoked potentials (MEPs) induced by transcranial magnetic stimulation (TMS) at 50 ms before surface-translation in each condition. The electromyographic (EMG) responses evoked by surface-translations were also measured. The results showed that the TA-MEP amplitude was greater in the High condition (where the largest TA-EMG response was evoked among the five conditions) compared to that in the No-perturbation, Low, and Posterior conditions (High vs. No-perturbation, *p* < 0.001; High vs. Low, *p* = 0.001; High vs. Posterior, *p* = 0.001). In addition, the MEP amplitude in the Random condition was significantly greater than that in the No-perturbation and Low conditions (Random vs. No-perturbation, *p* = 0.002; Random vs. Low, *p* = 0.002). The EMG response in the TA evoked by perturbation was significantly smaller when a perturbation can be predicted (predictable vs. unpredictable, *p* < 0.001). In the SOL and MG muscles, no prominent modulations of the MEP amplitude or EMG response were observed, suggesting that the effects of prediction on corticospinal excitability differ between the dorsiflexor and plantar flexor muscles. These findings suggest that the corticospinal excitability in the TA is scaled in parallel with the prediction of the direction and magnitude of an upcoming perturbation in advance.

## Introduction

Postural response to unexpected perturbation is a fundamental mechanism that functions to prevent falling in daily activities. The long-latency electromyographic (EMG) response, which contributes to the rapid correction of postural imbalance, is sensitive to the predictability of upcoming perturbation (Horak et al., 1989). Intriguingly, the effects of predictions vary depending on which factor can be known beforehand. When the timing of a perturbation’s onset can be predicted, the EMG amplitude is decreased despite the application of the same perturbation (Fujio et al., 2016). Prediction of the magnitude of a perturbation tunes the EMG amplitude for the appropriate response (Horak et al., 1989; Beckley et al., 1991), whereas prediction of the direction of a perturbation has no effect (Diener et al., 1991).

The responsiveness of the EMG amplitude is thought to be reflected in a presetting of the excitability of the neural system for postural control. The corticospinal pathway is one of the candidates for prediction-related EMG modulation. The excitability of this pathway in the tibialis anterior (TA) muscle and the soleus (SOL) muscle is modulated according to the posture state. Not only when changing position from supine to standing but also when maintaining a posture under unstable environments (such as standing on a foam surface; Papegaaij et al., 2014), the corticospinal excitability is enhanced as the posture must be balanced (Obata et al., 2009; Baudry et al., 2015). The corticospinal excitability can also be enhanced when a postural perturbation is imminent, even though the posture is not actually perturbed (Walchli et al., 2017; Fujio et al., 2018). Notably, this modulation is more pronounced when the timing of the onset of a perturbation is known, which is consistent with the results of investigations of the long-latency response (Ackermann et al., 1991; Fujio et al., 2016).

Given that the modulation of the corticospinal excitability represents the presetting of the neural state for an adequate postural response, the pattern of its change in corticospinal excitability would be tied to the magnitude and direction of an upcoming perturbation. However, little is known about whether the corticospinal excitability in the postural muscles is modulated with the prediction of these factors (i.e., the magnitude and direction of the perturbation). Electroencephalography (EEG) studies have demonstrated that a preparatory cortical activity before perturbation is scaled in parallel with the magnitude of the perturbation, and that the timing cue-related change in EEG was correlated with the difference in the center-of-pressure (COP) displacement (Jacobs et al., 2008; Mochizuki et al., 2010; Smith et al., 2012). If these changes in the cortical activities depend in part on the corticospinal pathway, the excitability of this pathway could vary depending on the prediction of the magnitude and direction of perturbations.

To address this question, we explored how the corticospinal excitability in the TA, SOL, and medial gastrocnemius (MG) muscles was modulated before three surface-translations that differed in respect to velocity, amplitude, and direction. In the present study, we defined “prediction” as an explicit and deterministic representation of the short-term future (Bubic et al., 2010). We measured both the motor-evoked potential (MEP) before postural perturbation and the EMG response after postural perturbation to examine whether the corticospinal excitability is modulated in parallel with the subsequent EMG response.

Based on the findings of previous EEG studies (Jacobs et al., 2008; Mochizuki et al., 2010; Smith et al., 2012), we hypothesized that the prediction of a larger magnitude of perturbation would induce a more pronounced enhancement of corticospinal excitability, and that the prediction of a smaller magnitude of perturbation would induce a less pronounced enhancement of corticospinal excitability. We focused on the TA rather than the SOL and MG because the postural response (i.e., the long-latency response) in the TA was markedly modulated in accord with the prediction of perturbation in our previous study, whereas no such modulation was observed in the SOL or MG (Fujio et al., 2016). We conducted the present study to determine whether the corticospinal excitability in the ankle muscles is specific to the predictions about the magnitude and the direction of perturbations. Our experiments’ results aimed to further the understanding of recent finding that the prediction of future events related to postural stability is a key modulator of corticospinal excitability (Walchli et al., 2017; Fujio et al., 2018).

## Materials and Methods

### Subjects

Twelve healthy volunteers (age 27.3 ± 1.6 years, all males) with no history of orthopedic, neurological, or psychiatric disorders participated. All subjects provided informed consent in accordance with the Declaration of Helsinki before the experiments, and all of the study’s protocols were approved by the Ethics Review Committee for Experimental Research with Human Subjects of the Graduate School of Arts and Sciences, the University of Tokyo.

### Experimental Setting

Anterior and posterior surface translations were applied using a movable platform: a six-degrees-of-freedom motion platform system actuated by an electric servomotor (Motion Base MB-150, Cosmate, Tokyo). A force plate (EFP-S-1.5kNSA13, Kyowa, Tokyo) was attached to this platform to monitor the COP during trials. Before the experiment, the subject freely selected the position of his feet on the force plate, and the selected position was traced with tape and a marker pen to be used for that subject in all trials. The subject was instructed to stand quietly on the force plate and to not move his head and limbs intentionally during the trials.

A mean COP position was measured at the beginning of the experiment for each subject, and it was used as a target area for the COP position which was 1 × 1 cm throughout the experiment. To enable each subject to maintain the same posture consistently among trials, the target COP area was displayed on a monitor placed 1.5 m in front of the subject. The ground reaction forces were recorded at a 4-kHz sampling frequency and low-pass filtered at 10 Hz (fourth-order zero-lag Butterworth filter).

In this study, the force data were used only for the display of the real-time COP position and were not analyzed further. Each subject underwent five trials for practice before every condition in order to enable them to keep their feet in place against perturbations. All subjects were able to compensate their standing posture without any steps within the practice period. We tracked the time courses of a single trial and computed the COPs with LabVIEW software (National Instruments, Austin, TX, USA).

### EMG

Surface EMG signals were recorded from the lower leg muscles, i.e., the TA, the SOL, and MG muscles. Bipolar electrodes (Ag-AgCl, 7-mm diameter) were placed 1.5 cm apart on the muscle belly and wrapped with thin elastic bandages to hold the electrodes and lead-lines. The EMG signals were amplified with a bioelectric amplifier (MEG-6108 bioelectric amplifier, Nihon Kohden, Tokyo) and band-pass filtered from 15 to 1,000 Hz. All signals were digitized at a sampling rate of 4 kHz.

The M-max of the SOL and the TA were obtained by electrical stimulation (a 1-ms rectangular pulse) of the posterior tibial nerve and the common peroneal nerve, respectively, for normalization of the EMG signals. For the stimulation of the posterior tibial nerve, the cathode was placed in the popliteal fossa and the anode was placed on the patella. The common peroneal nerve was stimulated below the neck of the fibula and the outer edge of the popliteal fossa. The stimulus intensity was increased gradually below the threshold level until the M-max was no longer increased.

### Transcranial Magnetic Stimulation (TMS)

Single-pulse transcranial magnetic stimulation (TMS) was delivered over the subject’s left motor cortex with a Magstim-200 stimulator (Magstim 200, Magstim Co., Whitland, UK) with a 110-mm double-cone coil. First, the optimal stimulus site at which the largest MEP of the TA could be evoked was searched for with the subject in a sitting posture. The location of the coil was drawn on the subject’s head with a marker pen to ensure a consistent position. Second, the active motor threshold (×1.0 MT) was measured with the subject in a standing posture as the minimal intensity for eliciting the peak-to-peak amplitude in the TA exceeding 50 μV in 5 of 10 consecutive stimulations. The coil position was monitored by a custom-built navigation system using a 3D motion capture system (Optitrack V100:R2, Natural Point, Corvallis, OR, USA; sampling 100 Hz).

To determine the coil position relative to the subject’s head, three reflective markers for the subject’s head and three reflective markers for the coil were attached. A single examiner who carried out all experiments guided the coil position behind the subject based on the navigation system and constantly supported the coil weight. The stimulus intensity was set at 120% of the MT of each subject. The MEPs of all three muscles were measured simultaneously by the single pulse at the scalp position defined by the above procedure. The TMS stimulation was delivered at 50 ms before the perturbation onset.

### Experimental Conditions

**Figure 1:**
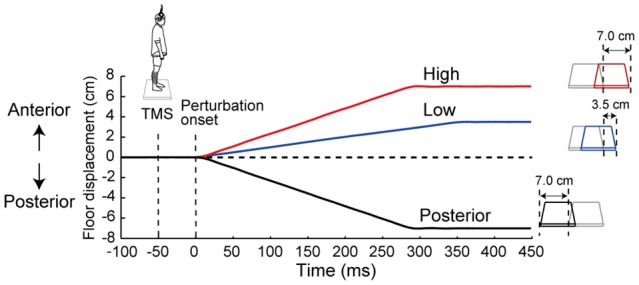
The time course of the floor displacement of three perturbations and transcranial magnetic stimulation (TMS) in a single trial. Three waveforms show the floor displacement in the High, Low and Posterior conditions, i.e., the Anterior-large, Anterior-small, and Posterior-large perturbations, respectively. “0 ms” corresponds to the beginning of the floor movement. TMS was applied 50 ms before the start of each perturbation.

To investigate the effects of the prediction of the magnitude and the prediction of the direction of the perturbation on the corticospinal excitability in the ankle muscles, we used three different perturbations in this study: two anterior translations (Anterior-large: 7.0 cm, 25.0 cm/s; Anterior-small: 3.5 cm, 10.0 cm/s) and one posterior translation (Posterior-large: 7.0 cm, 25.0 cm/s, Figure 1). The experiment consisted of five conditions based on these perturbation parameters and their order: (1) the No-perturbation condition in which no perturbation was applied; (2) the Low condition consisted of the Anterior-small perturbation; (3) the High condition was the Anterior-large perturbation; (4) the Posterior condition was the Posterior-large perturbation; and (5) the Random condition consisted of all three perturbations in randomized order. Two levels of perturbation magnitude were set only in the anterior translation because the TA was the primary target in this study. In the Random condition, the three perturbations types were distributed with equal probability. Thus, perturbations in the Random condition were unpredictable while ones in the Low, High and Posterior conditions were predictable.

A total of 15 trials were carried out for each condition: TMS was applied in 10 of 15 trials (MEP trial), and not applied in the other 5 out of the 15 trials (catch trial). The order of these trials was randomized to avoid order effects. In the catch trial, the subject was perturbed with the TMS coil kept in place on his head as the MEP trials. The objective of the catch trials was to assess the EMG responses evoked by perturbations without any effects of TMS (i.e., TMS-evoked activation of the motor cortex). In the random condition of the 10 MEP trials, two out of the three perturbation types (Anterior large, Anterior small, and Posterior large, selected randomly) were each administered three times, and one out of the three perturbation types was done two times. In the random condition of five catch trials, two out of three perturbation types were given two times respectively, and one out of three perturbation types was done one time. The order of the five conditions was randomized, and the subjects were told which condition would be performed in the following set. Five practice trials were carried out before each condition to familiarize the subject with the perturbation and to ensure an appropriate presetting corresponding to the upcoming perturbation. An acoustic cue was provided 1.0 s before the onset of the perturbation to encourage the presetting of the corticospinal excitability before perturbation.

### Data Analysis and Statistical Analyses

We calculated the peak-to-peak amplitude of each MEP within 40 ms after stimulus onset and compared the amplitudes among the five conditions. We set a reference value as deviation above 1.5× the interquartile range (IQR) from the 3rd-quartile and below 1.5× the IQR from the 1st-quartile as an outlier (Fujio et al., 2018). After the outliers were discarded, we compared the averaged MEP amplitude among the five conditions. The root mean square (RMS) of background EMG activity (BGA) was computed for 50 ms before the TMS stimulation. We discarded the trials in which the RMS of the BGA exceeded the mean value plus two times its standard deviation (BGA + 2 SD) of all trials in the same condition. All MEPs were normalized by the M-max of each muscle.

In the catch trials, the integrated EMG (iEMG) activity was calculated to confirm the difference in the evoked EMG activities depending on the perturbations. After the rectification and subtraction of the DC bias, we normalized the EMG activities using the averaged amplitude from the perturbation onset to 250 ms post-onset in the High condition for the TA and in the Posterior condition for the SOL and MG. The iEMG was calculated for 100 ms from the time period defined by the onset of the ensemble-averaged EMG response in the High condition for the TA and in the Posterior condition for the SOL and the MG. To determine the EMG onset in the two conditions, the horizontal line at the level of the mean BGA plus its 2 SD in each trial was depicted on the computer monitor, and the timing that was over the horizontal line was confirmed. When the EMG amplitude was higher than this reference for 10 ms, the time period was defined as the latency of the EMG response. The iEMG averaged in all catch trials was compared among the Low, High, and Posterior conditions to confirm the required EMG response for each perturbation.

In the Random condition, the three different perturbations, (Anterior-large, Anterior-small and Posterior-large) were mixed in randomized order. Both the MEP amplitude and the iEMG were averaged respectively in the three different perturbations. The iEMG evoked by the Anterior-large, Anterior-small and Posterior-large perturbations was compared with that in the High, the Low and the Posterior conditions in which the corresponding perturbation was applied.

We performed a repeated one-way analysis of variance (ANOVA) on the values of the averaged peak-to-peak MEP amplitude, the BGA, and the iEMG. When sphericity could not be assumed, a Greenhouse-Geisser correction was performed. For a multiple comparison analysis, we used a Bonferroni corrected *post hoc* test for all pairwise comparisons (*α* = 0.005). For the comparison of the iEMG of each perturbation in the Random condition with that of the corresponding iEMG in the Low, High, and Posterior conditions, a two-way ANOVA (predictability × perturbation) was performed to reveal the effect of prediction on the EMG response. The significance level for all statistical tests was set at 0.05. Partial eta squared values were calculated as the effect size for ANOVA. The data are presented as mean ± standard error (SE).

## Results

**Figure 2:**
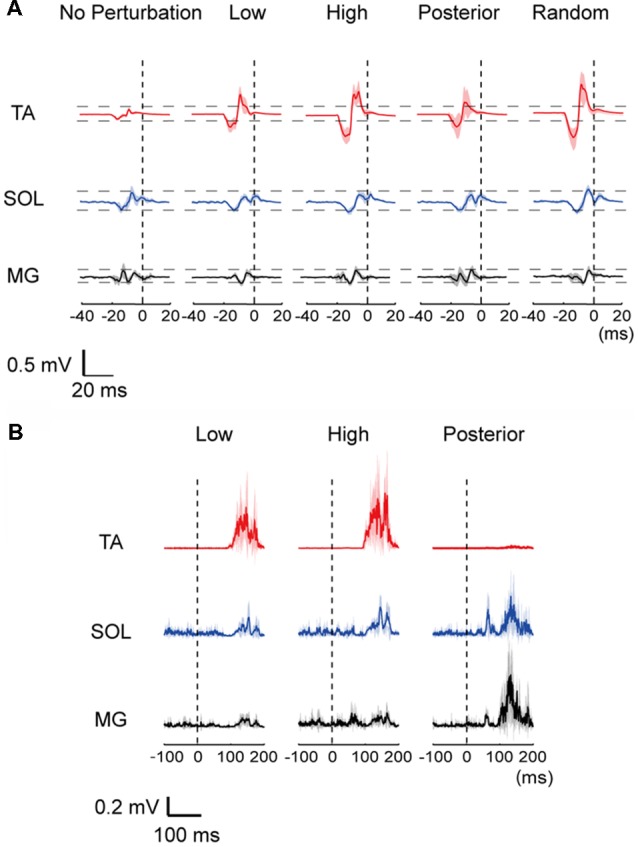
**(A)** Representative row motor-evoked potentials (MEPs) imposed for all trials for one subject. The MEPs in the three muscles are depicted at the same scale. “0”: the time point of perturbation onset. TA, tibialis anterior; SOL, soleus; MG, medial gastrocnemius. **(B)** Muscle activities of the TA, SOL and MG following a perturbation in a representative data. A mean (thick line) and standard error (SE; shaded) activities in each muscle were depicted. “0 ms” corresponds to the beginning of the floor movement.

All subjects were able to become accustomed to the experimental protocol in their practice trials and completed the experiment without falling. Representative MEP traces and EMG responses with all waveforms of one subject superimposed are provided in Figure 2. The amplitude of the TA-MEP tended to be large in the order of the High, Random, Low, Posterior, and No-perturbation conditions. In the SOL and MG, the MEP amplitude did not differ clearly among the five conditions (Figure 2A). The EMG responses evoked by perturbation were clearly different between the ankle dorsiflexor and plantar flexor muscles: larger TA responses were induced with the anterior translations (the Low and High conditions) and larger SOL and MG responses were induced with the posterior translation (Figure 2B).

**Figure 3:**
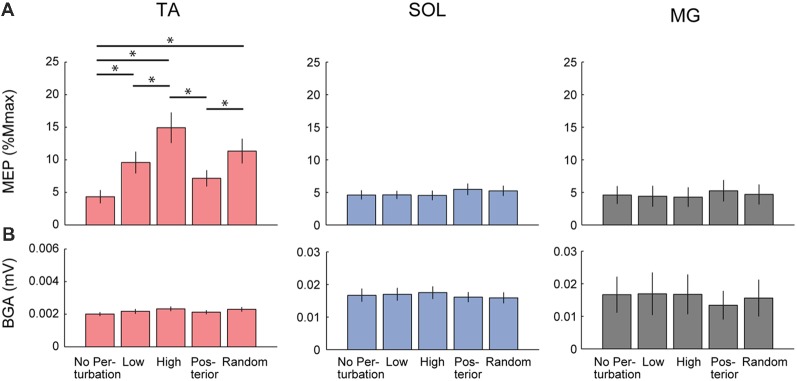
Comparison of the Group average of the MEP **(A)** and the background EMG activity (BGA; **B**) in each muscle among the five conditions. Significant differences were observed in the TA-MEP. TA, tibialis anterior; SOL, soleus; MG, medial gastrocnemius. Asterisks indicate the statistically significant differences between conditions.

For the group data, the one-way repeated ANOVA for the TA-MEP revealed the significant main effect of condition (*F*_(2.30,25.3)_ = 15.15, *p* < 0.001, *η*^2^ = 0.579, Figure 3A). The *post hoc* comparison demonstrated that the amplitude of the TA-MEP in the Low, High, and Random conditions was significantly larger than that in the No-perturbation condition (No-perturbation vs. Low, *p* = 0.001; No-perturbation vs. High, *p* < 0.001; No-perturbation vs. Random, *p* = 0.002). Moreover, the TA-MEP in the High condition was significantly higher compared to that in both the Low and Posterior conditions (High vs. Low, *p* = 0.001; High vs. Posterior, *p* = 0.001). In the Random condition, the TA-MEP was also significantly larger than that in the Posterior condition (*p* = 0.002). There was no significant difference in the TA-MEP between the Low and Posterior conditions.

**Figure 4:**
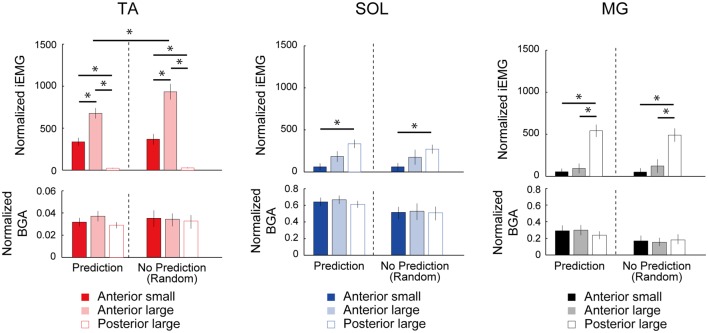
Comparison of the normalized integral electromyographic (EMG) values for 100 ms post-EMG onset between the predictable (Low, High, Posterior) and unpredictable (Random) conditions. The TA-EMG in the Anterior-large perturbation was significantly different between the High and Random conditions. In the comparison of the three types of perturbations, there were significant differences of integrated EMG (iEMG) in all three muscles depending on the perturbation direction. The BGA activities were not significantly different among the three types of perturbation. TA, tibialis anterior; SOL, soleus; MG, medial gastrocnemius. Asterisks indicate the statistically significant differences between conditions.

In the SOL, there was also a significant main effect of condition (*F*_(4,44)_ = 2.93, *p* = 0.03, *η*^2^ = 0.210, Figure 3). However, the *post hoc* comparison using Bonferroni correction did not detect any significant difference in the SOL-MEP among the five conditions. Regarding the MG, a main effect was not observed in the MG-MEP (*F*_(4,44)_ = 2.15, *p* = 0.09, Figure 3). The BGAs were not significantly different among the five conditions in all three muscles (TA, *F*_(1.92,21.15)_ = 3.49, *p* = 0.05; SOL, *F*_(4,44)_ = 0.52, *p* = 0.72; MG, *F*_(4,44)_ = 0.66, *p* = 0.63). In the Random condition, we compared the MEP amplitude before the three respective perturbations. The results showed that there was no significant difference among the different perturbations in the TA-MEP, demonstrating that when the magnitude and direction of perturbation could not be predicted, the MEP was larger compared to that in normal standing, and the enhancement that was matched to the perturbation had disappeared (TA, *F*_(18,2)_ = 0.82, *p* = 0.46; SOL, *F*_(18,2)_ = 1.03, *p* = 0.38; MG, *F*_(10.53,1.17)_ = 0.31, *p* = 0.63). Figure 4 presents the iEMG of the ensemble-averaged EMG response evoked by no-TMS trials in the High, Low, and Posterior conditions. There were significant main effects of predictability on the iEMG in the TA (predictability, *F*_(10.00,1.00)_ = 40.82, *p* < 0.001, *η*^2^ = 0.803) and perturbation on the iEMG in all three muscles (TA, *F*_(10.81,1.08)_ = 169.77, *p* < 0.001, *η*^2^ = 0.944; SOL, *F*_(4.00,2.00)_ = 9.37, *p* = 0.031, *η*^2^ = 0.824; MG, *F*_(3.00,1.00)_ = 180.20, *p* = 0.001, *η*^2^ = 0.999). The *post hoc* comparison showed that the TA-iEMG in the Prediction condition was significantly smaller than that in the No Prediction (predictable vs. unpredictable, *p* < 0.001). In addition, the TA-iEMG was significantly different in all three pairwise comparisons (Anterior large vs. Anterior small, *p* < 0.001, Anterior small vs. Posterior large, *p* < 0.001, Anterior small vs. Posterior large, *p* = 0.001).

In the SOL and MG, significant differences were found in a *post hoc* comparison (SOL, Anterior large vs. Anterior small, *p* = 0.007; MG, Anterior large vs. Posterior large, *p* < 0.001; MG, Anterior small vs. Posterior large, *p* < 0.001). These results clearly demonstrated that the different direction of perturbation gave rise to different postural responses, and the predictability affected the TA response. The latency of the TA response in Anterior large (i.e., the High condition) was 114.4 ± 7.0, and the latencies of the SOL and MG in Posterior large (i.e., the Posterior condition) were 110.7 ± 11.3, and 109.5 ± 5.9, respectively.

## Discussion

The results of this study demonstrated that the corticospinal excitability in the TA was modulated depending on the direction and amplitude of predictable perturbation. The TA-MEP was more prominent when anterior translation was applied compared to when posterior translation was applied. In addition, when the surface translation was to the anterior direction, the TA-MEP was greater when the amplitude of the translation was expected to be large than when it was expected to be small. These findings suggested that the corticospinal excitability in the TA was scaled in parallel with the prediction of the direction and magnitude of an upcoming perturbation in advance. On the other hand, no modulation of MEPs among conditions was observed in the SOL or the MG. The difference between the ankle dorsiflexor and plantar flexor muscles may reflect a differential neural control for postural disturbance, probably due to differences in the functional roles and the strength of the corticospinal input between the TA and SOL muscles.

### Cortical Involvement in the Presetting of the Corticospinal Excitability According to Prediction

Corticospinal excitability is known to be modulated at both supraspinal and spinal levels (Nielsen et al., 1993). It is possible that the excitability of neurons and interneurons at the motor cortex and spinal cord and their synaptic transmissions are attributable to the modulation of corticospinal excitability. However, based on the findings of EEG studies (Jacobs et al., 2008; Mochizuki et al., 2010), we suspect that primarily the motor-related area in the cortex contributes to our present results.

EEG studies revealed that the predictability of the perturbation magnitude was associated with preparatory cortical activity called the “contingent negative variation (CNV)” at the Cz electrode (Jacobs et al., 2008; Mochizuki et al., 2010). When the perturbation was imminent, the CNV evoked by an auditory signal was increased compared to that in normal standing, and its increments tended to be smaller when a larger perturbation was predicted. In addition, when the magnitude of perturbation was unknown, the CNV was also increased. These findings suggested that the motor-related cortical activities were preset by the prediction of the magnitude of a perturbation and of its occurrence. A more recent study further revealed that the prediction of direction and magnitude modulated the perturbation-evoked EEG response which reflected the sensory processing (Goel et al., 2018). Both preparatory and post-perturbation EEG activities are simultaneously changed based on a predicted perturbation.

The present results are consistent with these reports in regard to a clearer enhancement of the corticospinal excitability in the TA before both larger and unknown perturbations. The changes in the motor-related cortical activities demonstrated by EEG may include the modulation of the corticospinal excitability. A higher nervous center, such as the supplementary motor area and the prefrontal cortex, would be a candidate as a modulator for this pathway, since those areas are involved in balance reactions (Mihara et al., 2008; Marlin et al., 2014).

### Implications of the Presetting of the Corticospinal Excitability for EMG Response

The present results demonstrate that the TA-MEP was scaled with the prediction of the magnitude of an upcoming perturbation in advance. This change in the corticospinal excitability would play a role in the modulation of postural responses depending on predictable perturbation, since the corticospinal pathway is one of the important pathways for the long-latency EMG response (Petersen et al., 1998; Taube et al., 2006). The classical studies of postural responses demonstrated that EMG responses are scaled with the prediction of the magnitude of an upcoming perturbation (Horak et al., 1989; Beckley et al., 1991; Horak and Diener, 1994). When a larger magnitude of perturbation is predicted, higher responsiveness is set in advance, and when a smaller magnitude of perturbation is predicted, lower responsiveness is set in advance. The present results suggest that the corticospinal pathway is likely to provide the underlying neural mechanism to scale the TA-EMG response described in the above-mentioned studies.

Our findings also demonstrated that when the perturbation direction could be predicted, the enhancement in the TA excitability varied based on the direction: the excitability was significantly enhanced before the anterior-surface translation but not before the posterior-surface translation. This result seems reasonable regarding functional relevance because the TA-EMG response primarily stabilizes the ankle joint accompanied by posterior body sway. However, it has been reported that the spatial prediction regarding the perturbation direction does not affect the EMG response (Diener et al., 1991; Fujio et al., 2016). A question thus arises: why is the TA cortical excitability modulated depending on spatial prediction?

One of the possible explanations is that the presetting of the TA reflects EMG patterns of voluntary movements after an automatic postural response. It was reported that the spatiotemporal EMG patterns of upper limb voluntary movement are represented at the motor-related cortical area (Gentner and Classen, 2006; Overduin et al., 2015). Therefore, even though spatial prediction does not affect the EMG response, it is not meaningless but rather can be used to facilitate voluntary activation of the TA.

### The Influence of the Unpredictability of the Magnitude and Direction of Upcoming Perturbation

When different intensities of surface translations are applied in randomized order, the EMG responses are tuned for the intermediate size of potential perturbations (Horak et al., 1989). Interestingly, this is not restricted to the case in which a large perturbation that suggests a threat of falling is included. In this situation, the EMG responses are estimated for the largest size of the predicted perturbations (Beckley et al., 1991). The present TA-MEP results showed that the amplitude in the random condition was around an intermediate value of that in the three different perturbations (Low: 9.7 ± 1.6% M-max, High: 14.8 ± 2.3% M-max, Posterior: 7.3 ± 1.2% M-max, Random: 11.5 ± 1.9% M-max), which supports the results of the Beckley et al. (1991) study. This is because the largest perturbation in our experiment was smaller in regard to the displacement and the velocity compared to the Horak et al. (1989) study. Compared to the situation in normal standing, the presetting of the corticospinal excitability was tuned on a higher level in the situation of unpredictable magnitude and direction of a perturbation, which is similar to the findings of EEG studies (Mochizuki et al., 2010; Smith et al., 2012). These results support the idea that the predictions contribute to the presetting of the appropriate level of the corticospinal excitability.

We also observed effects of unpredictability on the size of the TA-EMG response. The unpredictable Anterior-large perturbation induced a significantly larger TA response compared to the predictable perturbation. Given that the corticospinal excitability tended to be set at a lower level in the unpredictable condition (High vs. Random, 14.8 ± 2.3% M-max vs. 11.5 ± 1.9% M-max), there was a mismatch between the presetting level and the actual perturbation in the Random condition. We suspect that this estimation error in the forward model caused the larger EMG response compared to the predictable condition (Scott, 2004). For a more direct elucidation of the causal relationship, future studies should investigate whether the modulation of the pre-corticospinal excitability is compatible with the post-EMG amplitude, trial-by-trial.

### Different Modulation Between the Dorsiflexor and Plantar Flexor Muscles

In agreement with previous findings (Obata et al., 2009; Fujio et al., 2018), we observed that the TA excitability was susceptible to the prediction of a perturbation, whereas the SOL and MG excitabilities showed no change. Our observations confirmed that the enhancement of the plantar flexor muscles was lesser even though the posterior-surface translation to which those muscles activated as the prime mover was predicted. The different responsiveness between the dorsiflexor and plantar flexor muscles was probably due to differences in the strength of the cortical input.

The responses to TMS (i.e., MEPs) in both the SOL and the MG tend to be smaller (especially at high stimulus intensity) compared to those in the upper limb muscles, whereas the MEP in the TA is comparable (Brouwer and Ashby, 1990; Bawa et al., 2002; Oya et al., 2008). These differences were thought to reflect the poor corticospinal projections to the SOL and MG motoneurons. In addition, the net inputs including the excitatory and inhibitory projections could be different between dorsiflexor and plantar flexor muscles. If TMS activates mainly the inhibitory projections, which have a lower threshold than the excitatory projections (Nielsen and Kagamihara, 1993) and which are richer in the plantar flexor muscles (Hudson et al., 2013), then clear enhancement would not be observed in the SOL or MG.

In addition, we suspect that the BGA in the SOL and MG muscles during standing contributes to the subtle changes of the MEP. Theoretically, a low-active muscle can show a larger extent of modulation because of the number of motoneurons in the subliminal fringe (Capaday and Stein, 1987). This is supported by the findings that the modulation of the cortical activity tended to be weak during the preparatory period when the target muscle was active (MacKinnon et al., 2000; Lewis et al., 2006; Meziane et al., 2009; Pruszynski et al., 2014). Given that the TA is the “silent” muscle in normal standing, this may be one of the reasons why the TA has the potential for a remarkable change before perturbation.

### Study Limitations

The present results should be interpreted with some limitations. First, only one level of perturbation magnitude was set in the posterior direction (Posterior large), whereas two level of perturbation magnitude were set in the anterior direction (Anterior large vs. Anterior small). This was because we focused on the TA and because we confirmed that the TA was markedly modulated compared to the SOL and the MG in a pilot experiment. However, this limits the interpretation of our presentthe SOL- and MG-MEP findings. It remains unclear whether the SOL- and MG-MEPs would be scaled on the basis of the prediction of the perturbation magnitude, whereas no effect of the prediction of the perturbation direction was observed in these MEPs. The question of how plantar flexor muscles are modulated with prediction should be investigated more extensively.

Second, the participants in this study were all male. Although there is little difference in the stimulus-intensity relation of the MEP between young males and females (Wassermann, 2002; Pitcher et al., 2003), a possible sex difference appeared due to change in sex hormones with aging (Smith et al., 2011). Only young male adult participated in the present study, and the effect of a sex difference can be ignored.

## Conclusion

We investigated whether the corticospinal excitability in the TA, SOL, and MG muscles was scaled with the predictions of the magnitude and the direction of perturbations. Our results demonstrate that the prediction of both the magnitude and the direction of perturbations has effects on the corticospinal excitability in the TA. This implies that the predictive control of corticospinal excitability is one of the neural mechanisms underlying a postural response. It thus appears that the prediction is one of the key abilities in balanced standing. Future studies are expected to reveal the relationship between the modulation of the corticospinal pathway and the stability of standing posture.

## Author Contributions

KF designed the study, carried out all experiments and analysis, interpreted the results, and wrote the original draft. HO, NK and KN helped to design the study, participated in the interpretation of the results and corrected the manuscript. KN supervised the project.

## Conflict of Interest Statement

The authors declare that the research was conducted in the absence of any commercial or financial relationships that could be construed as a potential conflict of interest.
